# The association between motor coordination, behavior, cognition and brain structure in children: an ABCD study

**DOI:** 10.1007/s00787-025-02931-2

**Published:** 2026-04-06

**Authors:** Elena M. Bonke, Suheyla Cetin-Karayumak, Fan Zhang, Yorghos Tripodis, Caroline Seer, Michael Frey, Anja K. Betz, Philine Rojczyk, Leo R. Zekelman, Johanna Seitz-Holland, Steve Pieper, Gerd Schulte-Koerne, Martha E. Shenton, Lauren J. O’Donnell, Yogesh Rathi, Inga K. Koerte

**Affiliations:** 1 cBRAIN, Department of Child and Adolescent Psychiatry, Psychosomatics and Psychotherapy, University Hospital Ludwig-Maximilians-Universität, Munich, Germany; 2 University Hospital, Ludwig-Maximilians-Universität, NeuroImaging Core Unit Munich (NICUM), Munich, Germany; 3 German Center for Child and Adolescent Health (DZKJ), partner site Munich, Munich, Germany; 4 Psychiatry Neuroimaging Laboratory, Department of Psychiatry, Brigham and Women’s Hospital, Harvard Medical School, Boston, MA USA; 5 Department of Radiology, Brigham and Women’s Hospital, Harvard Medical School, Boston, MA USA; 6 Department of Biostatistics, Boston University School of Public Health, Boston, MA USA; 7 Alzheimer’s Disease and CTE Centers, Boston University School of Medicine, Boston, MA USA; 8 Movement Control and Neuroplasticity Research Group, Department of Movement Sciences, KU Leuven, Leuven, Belgium; 9 KU Leuven Brain Institute (LBI), KU Leuven, Flanders, Belgium; 10 Faculty of Applied Healthcare Sciences, Deggendorf Institute of Technology, Deggendorf, Germany; 11 Department of Child and Adolescent Psychiatry, Psychosomatics and Psychotherapy, University Hospital Ludwig-Maximilians-Universität, Munich, Germany; 12 Graduate Program in Speech and Hearing Bioscience and Technology, Harvard Medical School, Boston, MA USA; 13 Department of Neurosurgery, Brigham and Women’s Hospital, Harvard Medical School, Boston, MA USA; 14 Department of Psychiatry, Massachusetts General Hospital, Harvard Medical School, Boston, MA 02114 USA; 15 Isomics, Inc., Cambridge, MA USA; 16 VA Boston Healthcare System, Brockton Division, Brockton, MA USA

**Keywords:** Motor coordination, Behavior, Cognition, White matter microstructure

## Abstract

The prevalence of behavioral and cognitive difficulties in children is high and often remains undetected. Evidence suggests that poor motor coordination may be an early indicator of these difficulties. However, the underlying mechanisms are unknown. This study investigates whether motor coordination is associated with behavioral and cognitive outcomes, and whether white matter microstructure characteristics mediate these relationships. Data from 9342 children participating in the Adolescent Brain Cognitive Development (ABCD) study was analyzed. Children were categorized in two groups: *normal* (*n* = 8239; mean age = 9.93; 48.5% female) and *poor motor coordination* (*n* = 1103; mean age = 9.88; 42.3% female) based on parent-report. Behavior was assessed using the Child Behavior Checklist, cognitive functioning using the Youth National Institute of Health toolbox, and white matter microstructure using diffusion magnetic resonance imaging. Children with poor motor coordination had significantly more behavioral difficulties, lower cognitive functioning and altered white matter microstructure. White matter microstructure partly mediated the associations between motor coordination and behavioral and cognitive outcomes.These findings underline the importance of early identification of poor motor coordination to support children at risk of broader developmental concerns, including behavioral and cognitive difficulties.

**Supplementary Information:**

The online version contains supplementary material available at 10.1007/s00787-025-02931-2.

## Introduction

 Behavioral and cognitive difficulties are common in children and adolescents and often remain undiagnosed, leading to long-term negative consequences. Reasons for the lack of early detection are multifactorial. For instance, children and their caregivers may hesitate to seek professional help because they underestimate the need or because they may fear social stigma [1, 2]. Moreover, identifying such difficulties early may be challenging given that existing screening tools are not widely applied and access to specialized medical care may be limited. In fact, whether a child’s behavioral and cognitive difficulty is identified by a primary care physician often depends on the parents drawing the physician’s attention to observed issues [3]. If problems are being identified early, targeted support and, if necessary, therapeutic interventions may be provided to alleviate symptoms and to prevent adverse long-term outcome [4].

Emerging evidence suggests that poor motor coordination may serve as such an early indicator. Studies have shown that children with motor coordination difficulties are more likely to exhibit behavioral problems and lower cognitive performance [5–7]. Longitudinal data even suggest that early motor difficulties can predict later mental health outcomes [8]. The environmental stress hypothesis provides a theoretical framework, proposing that poor motor coordination may lead to psychosocial stressors such as peer rejection and low self-esteem, which in turn increase the risk of behavioral and emotional issues [9].

Despite this, the neurobiological underpinnings of the relationship between motor coordination, behavioral difficulties, and cognitive functioning, remain unknown. One approach to investigate the underlying neural mechanisms is the use of magnetic resonance imaging (MRI), or specifically diffusion magnetic resonance imaging (dMRI) to investigate potential alterations in white matter microstructure. dMRI objectively quantifies diffusion properties of water molecules in the white matter of the brain, and, thus, gives insight into the tissue microstructure of the brain including fiber orientation and directionality [10, 11]. Prior studies have linked motor function to the corticospinal tract/posterior limb of the internal capsule and cerebello–thalamo–cortical pathways, cognitive functions to the superior longitudinal fasciculus and corpus callosum, and behavioral/emotional outcomes to the cingulum and uncinate fasciculus [12–15].

The current study investigates whether poor parent-reported motor coordination in children is associated with behavioral and cognitive difficulties in a large cohort of children, and whether brain white matter microstructure mediates these associations. These white matter microstructure alterations may be viewed as an epiphenomenon of combined motor, behavioral and cognitive difficulties.

## Methods

### Participants

Data were drawn from the ABCD study. We used release 4.0 for all except the dMRI data, because data from release 3.0 was harmonized before data release 4.0 was available. Details on the ABCD study design were described by Garavan et al. (2018) [16]. Briefly, the ABCD study investigates behavior, cognition, and brain development amongst other aspects, in over 11,000 nine-to-ten-year-old children [16]. The baseline data of 11,876 participants aged nine-to-ten years were acquired between 2016 and 2018. All caregivers provided written informed consent, and all children provided verbal assent to participate in the study approved by institutional review boards.

### Demographic variables

Demographic measures include biological sex (male/female), age in years, BMI, handedness (right/ambidextrous/left), race (White/Black or African American/American Indian and Alaska Native/Asian/Native Hawaiian and other Pacific Islander/Multiple/Other), and total family income. BMI was calculated as follows: BMI = (weight in pounds x 703)/(height in inches x height in inches). Handedness was assessed using the Youth Edinburgh Handedness Inventory Short Form (EHIS) [17]. Total family income was reported as ordinal scale from 1 (*<$5K*) to 10 (*>$200K*) and was used as a proxy for socio-economic status.

### Motor coordination

Parent-reported motor coordination was based on item 62 of the Child Behavior Checklist (CBCL; abcd_cbcl01) [12]. For this item, the caregiver is asked to rate whether his/her child is *poorly coordinated or clumsy* with 0 = *not true*, 1 = *sometimes true*, or 2 = *very often true*. For this study, the answer *not true* indicated *normal motor coordination* (*n* = 8239) and the answers *sometimes* or *very often true* indicated *poor motor coordination* (*n* = 1103). Splitting the variable assessing normal/poor motor coordination in dichotomous outcomes has previously been performed to achieve higher diagnostic utility compared to when investigating differences between three groups [18].

### Behavior

Behavioral difficulties were assessed using the Child Behavior Checklist (abcd_cbcls01). The CBCL is one of the most widely investigated tools to detect emotional and behavioral difficulties in children and adolescents. Even though the CBCL is considered a screening tool, many studies have demonstrated a high classification overlap between CBCL outcomes and clinical diagnoses (for review see [19]). Of note, eight items of the CBCL including item 62 (poor motor coordination/clumsy) were previously found to be particularly predictive of psychiatric disorders [20]. The CBCL is based on parent-report and contains eight individual scores that can also be combined in three summary scores:

The *Externalizing Summary Score* is the sum of: Attention Problems, Rule-breaking, and Aggressive Behavior. The *Internalizing Summary Score* is the sum of: Anxious/Depressed, Withdrawn/Depressed, and Thought Problems. Finally, the *Total Summary Score* is the sum of Externalizing and Internalizing Score and the Somatic Score and Social Problem Score. Raw scores were transformed to norm scores by adjusting for age and sex. Higher scores indicate more behavioral difficulties.

Here, in addition to the use of norm scores, we apply a dichotomous group split to differentiate between the presence or absence of clinically meaningful problem behavior, an approach often used in clinical as well as research settings. Following this approach, a summary score ≥ 64 (equivalent of 98th percentile) or an individual score ≥ 70 (equivalent of 98th percentile) was defined as presence of behavioral difficulties [21].

### Cognitive functioning

Cognitive functioning was assessed using the ABCD Youth National Institute of Health toolbox (NIH TB) Summary Scores (abcd_tbss01) [22]. This assessment consists of seven validated and reliable psychometric tests:

The *Fluid Composite Score* combines Picture Sequence Memory Test, Flanker Inhibitory Control and Attention Test, List Sorting Working Memory Test, Dimensional Change Card Sort Test, and the Pattern Comparison Processing Speed Test. *The Crystallized Composite Score* combines Picture Vocabulary Test and the Oral Reading Recognition Test. The *Total Composite Score* combines all seven tests. For all tests, higher scores indicate better cognitive performance. Here, age-corrected norm scores were used where 100 indicates average performance and ± 15 indicating one standard deviation.

### Neuroimaging

White matter microstructure was computed using dMRI data collected from 10,866 participants. dMRI acquisition (1.7 mm^3^ isotropic resolution with 96 gradient directions and 4 b-value shells: 500, 1000, 2000, 3000 s/mm^2^) was performed at 21 sites on 45 different scanner settings using 3 T Siemens Prisma, Philips, and GE scanners. The details on the dMRI acquisition can be found in Hagler et al. [23].

#### Pre-processing and quality check of the data

The preprocessing steps including eddy and motion correction, b0 inhomogeneity correction, and gradient unwarp were completed by the ABCD study team. Similarly, the image quality of all dMRI raw datasets was visually inspected by trained reviewers from the ABCD study team [23]. Additional automated and manual quality control procedures were applied to ensure that dMRI scans were of good quality. The data from most GE and Siemens sites was of good quality, while data from Philips scanners contained artifacts (excessive spatial smoothing and motion artifacts) and was, thus, excluded from the harmonization process. The final harmonized diffusion MRI data consisted of 9345 participants obtained from 18 sites and 33 scanners.

#### Harmonization and processing of the dMRI data

Data acquired from different scanners/sites has large scanner specific effects that significantly reduce the statistical power of neuroimaging studies. Consequently, the data from different scanners must be harmonized (i.e., scanner related effects must be minimized or removed) before a pooled analysis of all data can be done. Diffusion MRI is particularly sensitive to scanner-related effects due to large differences in the way the sequence is played out on each scanner as well as the data reconstruction algorithms used. These scanner effects are highly nonlinear in nature and are best addressed in the acquired data rather than using linear statistical covariates during analysis of the derived data [24]. The baseline ABCD data was harmonized using our well validated harmonization algorithm [25] followed by running multi-fiber unscented Kalman Filter (UKF) tractography algorithm (https://github.com/pnlbwh/ukftractography*)* [26] and white matter anatomical fiber tract identification (https://github.com/SlicerDMRI/whitematteranalysis) [27] in conjunction with an anatomical white matter atlas [27] for automated extraction of 73 anatomical fiber tracts. Details about the quality control, the harmonization process and extraction of white matter measures can be found in Cetin-Karayumak et al. (2024) [28]. We note that since the dMRI data is harmonized at the beginning, no further statistical correction for study site is needed.

#### Extracting dMRI measures for white matter tracts

Multiple diffusion metrics of interests, including fractional anisotropy (FA; a putative measure of white matter fiber integrity), mean diffusivity (MD; a putative measure of total volume of diffusion), axial diffusivity (AD; a putative measure of the magnitude of diffusion parallel to fiber tracts), and radial diffusivity (RD; a putative measure of the magnitude of diffusion perpendicular to the fiber tracts) were extracted per subject for 73 white matter tracts, as part of the tractography parcellation process.

Based on preliminary group differences including all 73 tracts with left and right tract measures extracted separately, we found both hemispheres to be equally affected. Therefore, to reduce the number of investigated variables, in a second step, we combined left and right tracts for all tracts that were present in both hemispheres resulting in 40 investigated white matter tracts. Moreover, we decided to only report measures of FA, AD, and RD, as AD and RD allow for a more specific interpretation compared to MD. Furthermore, average FA, AD, and RD scores across all tracts were calculated and extracted.

### Statistical analysis

All statistical analyses were performed using the software R (version 4.0.1) [29] For transparency, results with covariates (*age*,* sex*,* study site*,* handedness*,* race*,* and family income*) are reported alongside results without including covariates. For analyses including data on cognitive functioning, age-corrected norm scores were used and thus, *age* was not included as covariate. For analyses including dMRI measures, since we included the harmonized dMRI data in our analysis, *study site* was not included as covariate. However, we corrected the dMRI measures for *body mass index (BMI)* because BMI was significantly different between groups and BMI has been shown to affect white matter diffusion measures/properties [30]. The significance level was set to *p* <.05. All results were corrected for multiple comparisons using the Bonferroni correction method. To do so, all *p*-values were multiplied by the number of calculated tests excluding the total/average score (i.e., by 10 for problem behavior, by 9 for cognitive functioning, and by 40 for white matter microstructure).

#### Demographical characteristics

Chi-Square tests were used to assess between-group differences between children with poor and normal parent-reported motor coordination in the categorical variables *sex*, *handedness*, and *race*. A Wilcoxon test was used to assess between-group differences in the ordinal variable *family income*. Independent *t*-tests were used to assess between-group differences in the metrical variables *age* and *BMI*.

#### Odds ratios

Odds Ratios (OR) were reported only for differentiating between the presence or absence of clinically meaningful behavioral difficulties as the CBCL is the only measure that offers clinically validated cut-off values for abnormal clinical functioning. Based on the group split with 1 indicating the presence and 0 indicating the absence of behavioral difficulties, Odds Ratios (OR) and adjusted Odds Ratios (AOR) with 95% confidence intervals were calculated using logistic regression analyses.

#### Group differences

Between-group differences in behavior, cognitive functioning, and white matter microstructure were assessed using AN(C)OVAs.

#### Mediation analysis

The R-package *mediation* was used to investigate a possible mediation effect of white matter microstructure on the relationship between poor parent-reported motor coordination and behavior or cognition, respectively. To reduce the number of investigated analyses, FA averaged across all tracts was included as a mediator and the Total Summary Score (problem behavior)/the Total Composite Score (cognitive functioning) as the respective outcome. The analyses were performed without including covariates, with nonparametric bootstrapping (1000 simulations) and, for cognition, on a reduced dataset of complete data (*n* = 9050).

## Results

### Cohort characteristics

Out of the 11,876 participating children in the ABCD study, 9342 children who completed both the CBCL and NIH cognitive assessments had neuroimaging data of acceptable quality. Importantly, participants included in this study (*n* = 9342) did not significantly differ from the overall ABCD participants (*n* = 11,876) regarding sex, age, BMI, study site, handedness, race, and total family income (all *p* >.05).

The study sample consisted of children with normal parent-reported motor coordination (*n* = 8239, mean age 9.93 years, 48.5% female) and children with poor parent-reported motor coordination (*n* = 1103, mean age 9.88 years, 42.3% female). Children with poor coordination were more likely to be younger, male, ambidextrous, Black, had a higher BMI, and lower total family income (Table 1).

**Table 1:** Cohort characteristics in groups with normal and poor parent-reported motor coordination

	Normal motor coordination (*n* = 8239)	Poor motor coordination (*n* = 1103)	
	Mean ± SD	Mean ± SD	Group differences Independent t-test
**Age**	9.93 ± 0.63	9.88 ± 0.62	t(9340) = 2.455, ***p*** **=.014**
**BMI**	18.67 ± 4.12	19.51 ± 4.79	t(9332) = −6.297, ***p*** **<.001**
	**%**	**%**	**X**^**2**^**/Wilcoxon**
**Sex**			X^2^ = 14.804, ***p*** **<.001**
Female	48.5%	42.3%	
Male	51.5%	57.7%	
**Handedness**			X^2^ = 13.567, ***p*** **=.002**
Right	80.4%	75.9%	
Left	7.0%	7.9%	
Ambidextrous	12.6%	16.2%	
**Race**			X^2^ = 24.275, ***p*** **<.001**
White	65.4%	59.1%	
Black	15.7%	19.0%	
Asian	1.8%	1.4%	
American Indian/Alaska Native	0.6%	0.9%	
Native Hawaiian/Pacific Islander	0.1%	0.4%	
Multiple	12.6%	15.0%	
Other Race	3.9%	4.3%	
**Total Family Income**			W = 4,395,868, ***p*** **<.001**
< $5K	3.5%	5.9%	
$ 5 K - $12K	3.6%	4.2%	
$12K-$16K	2.5%	2.9%	
$16K-$25K	4.4%	7.3%	
$25K-$35K	6.0%	7.8%	
$35K-$50K	8.6%	11.2%	
$50K-$75K	13.7%	13.4%	
$75K-$99K	14.6%	14.9%	
$100K-$199K	31.4%	24.0%	
> $ 200 K	11.9%	8.4%	

The variable race was re-coded based on guidelines provided by the National Institute of Health into the five minimum categories *White, Black or African American,American Indian or Alaska Native, Asian, Native Hawaiian or Other Pacific Islander*, as well as *Multiple and Other*^*70*^. Total family income is reported as ten ordinal categories ranging from *< $5K up to >$200K*.

### Group differences in behavior

**Fig. 1:**
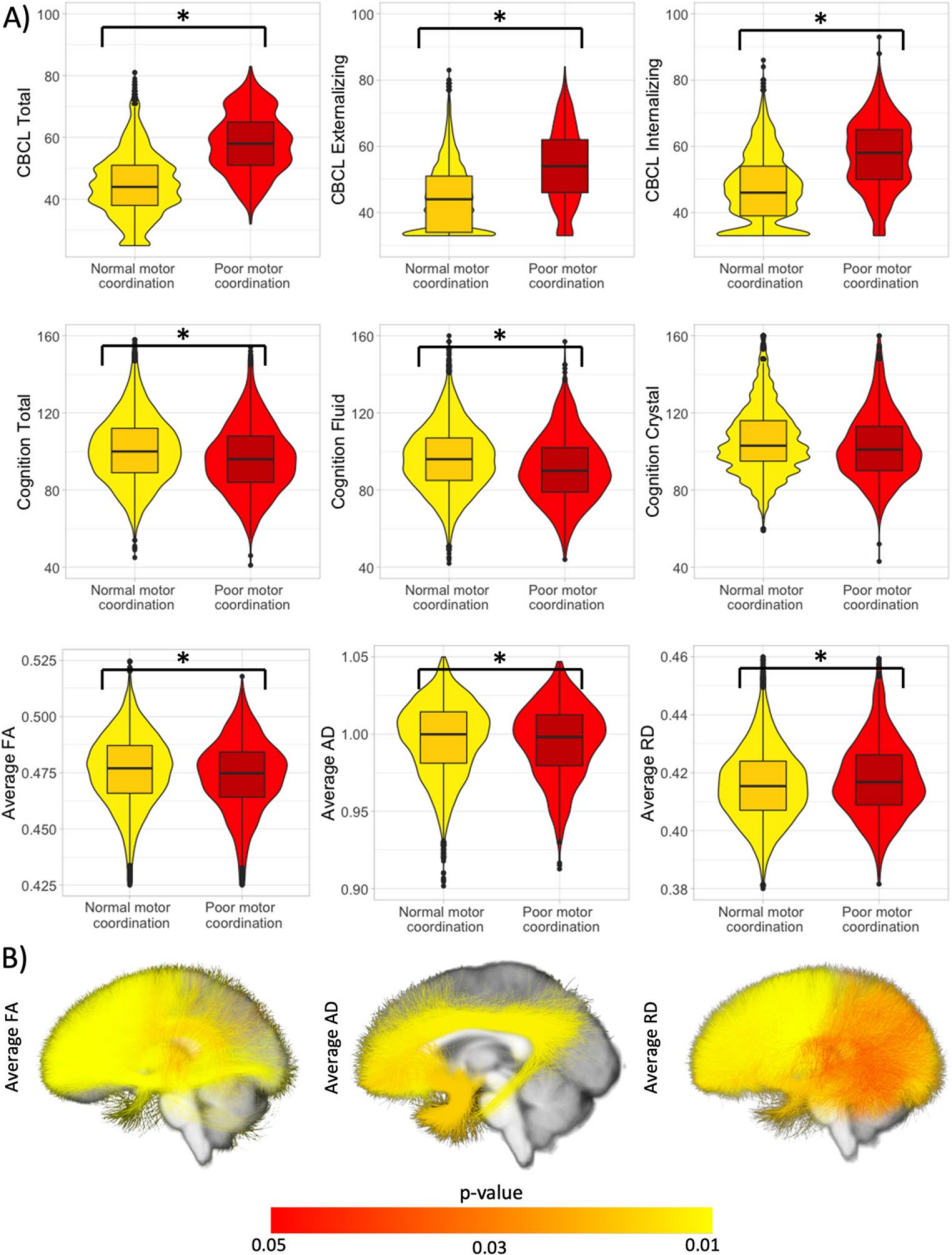
(**A**) Between-group differences between groups with poor and normal parent-reported motor coordination in behavior, cognition, and whole brain white matter microstructure. Higher values in the Child Behavioral Checklist (CBCL) indicate more behavioral difficulties. Higher scores in cognitive performance indicate better cognitive functioning, (**B**) Colored visualization (yellow-red continuum) of significance levels (p-values). P-values reflect the significance levels of group differences in white matter microstructure (fractional anisotropy, axial diffusivity, radial diffusivity) of fiber tracts between children with poor motor coordination and children with normal motor coordination after covariate and multiple comparison correction * Indicates statistical significance at *p* <.05 after covariate correction. Abbreviations. AD = axial diffusivity, FA = fractional anisotropy, RD = radial diffusivity

Between-group assessments (normal/poor parent-reported motor coordination) covarying for sex, age, study site, handedness, race, and total family income revealed statistically significant differences in all CBCL summary and all individual scores (all *p* <.001; Table 2 and Fig. 1). The group with poor motor coordination showed higher scores across all CBCL scores indicating more behavioral difficulties. Calculations with and without covariate corrections resulted in similar results. Calculations with and without counting item 62 in the Social Problems score resulted in similar results.

**Table 2:** Group differences between children with parent-reported normal and poor motor coordination in behavior, cognition and white matter microstructure

	Normal motor coordination (*n* = 8239)	Poor motor coordination (*n* = 1103)		
	Mean ± SD	Mean ± SD	Group differences with covariates	Group differences without covariates
**Behavior**				
**Total**	44.35 ± 10.56	58.05 ± 9.79	*F(1*,* 8425)* = 1453.32, ***p*** **<.001**, η² = .138	*F(1*,* 9340)* = 1668.10, ***p*** **<.001**, η² = .151
**Externalizing**	44.74 ± 9.74	53.89 ± 11.13	*F(1*,* 8425)* = 677.79, ***p*** **<.001**, η² = .070	*F(1*,* 9340)* = 829.05, ***p*** **<.001**, η² = .082
Attention	53.03 ± 4.96	60.66 ± 9.43	*F(1*,* 8425)* = 1529.78, ***p*** **<.001**, η² = .148	*F(1*,* 9340)* = 1758.50, ***p*** **<.001**, η² = .158
Aggression	52.28 ± 4.81	57.14 ± 8.33	*F(1*,* 8425)* = 651.64, ***p*** **<.001**, η² = .068	*F(1*,* 9340)* = 805.01, ***p*** **<.001**, η² = .079
Rule-breaking	52.35 ± 4.38	56.29 ± 7.00	*F(1*,* 8425)* = 550.05, ***p*** **<.001**, η² = .057	*F(1*,* 9340)* = 664.55, ***p*** **<.001**, η² = .066
**Internalizing**	47.33 ± 10.11	57.39 ± 10.65	*F(1*,* 8425)* = 826.93, ***p*** **<.001**, η² = .085	*F(1*,* 9340)* = 951.11, ***p*** **<.001**, η² = .092
Depressed	52.95 ± 5.36	57.86 ± 8.45	*F(1*,* 8425)* = 597.22, ***p*** **<.001**, η² = .064	*F(1*,* 9340)* = 694.36, ***p*** **<.001**, η² = .069
Withdrawn	52.90 ± 5.09	58.24 ± 8.39	*F(1*,* 8425)* = 739.01, ***p*** **<.001**, η² = .076	*F(1*,* 9340)* = 889.20, ***p*** **<.001**, η² = .087
Thought	53.11 ± 5.11	59.66 ± 8.35	*F(1*,* 8425)* = 1158.01, ***p*** **<.001**, η² = .116	*F(1*,* 9340)* = 1339.10, ***p*** **<.001**, η² = .125
Social	51.90 ± 3.46	59.60 ± 7.10	*F(1*,* 8425)* = 3098.24, ***p*** **<.001**, η² = .255	*F(1*,* 9340)* = 3493.00, ***p*** **<.001**, η² = .272
Somatic	54.44 ± 5.65	58.66 ± 7.58	*F(1*,* 8425)* = 431.51, ***p*** **<.001**, η² = .047	*F(1*,* 9340)* = 495.40, ***p*** **<.001**, η² = .050
**Cognition**				
**Total**	101.07 ± 17.62	96.52 ± 18.06	*F(1*,* 8161)* = 26.50, ***p*** **<.001**, η² = .003	*F(1*,* 9048)* = 62.844, ***p*** **<.001**, η² = .007
**Crystal**	105.90 ± 18.05	103.41 ± 18.46	*F(1*,* 8191)* = 2.06, *p* >.999, η² < .001	*F(1*,* 9083)* = 18.057, ***p*** **<.001**, η² = .002
Pic Vocab	107.07 ± 16.68	105.85 ± 17.75	*F(1*,* 8334)* = 0.81, *p* >.999, η² < .001	*F(1*,* 9237)* = 5.039, *p* =.223, η² < .001
Reading	102.91 ± 18.93	99.97 ± 18.71	*F(1*,* 8320)* = 7.81, ***p*** **=.047**, η² < .001	*F(1*,* 9229)* = 23.25, ***p*** **<.001**, η² = .003
**Fluid**	96.34 ± 17.02	91.20 ± 17.50	*F(1*,* 8657)* = 48.28, ***p*** **<.001**, η² = .005	*F(1*,* 9052)* = 85.696, ***p*** **<.001**, η² = .009
Picture	101.55 ± 16.19	98.29 ± 15.24	*F(1*,* 8329)* = 18.59, ***p*** **<.001**, η² = .002	*F(1*,* 9231)* = 39.515, ***p*** **<.001**, η² = .004
Flanker	95.92 ± 13.43	93.67 ± 14.33	*F(1*,* 8329)* = 17.08, ***p*** **<.001**, η² = .002	*F(1*,* 9231)* = 26.708, ***p*** **<.001**, η² = .003
List	101.14 ± 14.53	98.27 ± 15.42	*F(1*,* 8313)* = 18.61, ***p*** **<.001**, η² = .002	*F(1*,* 9210) =* 36.824, ***p*** **<.001**, η² = .004
Card sorting	97.26 ± 15.12	94.08 ± 14.82	*F(1*,* 8332)* = 20.56, ***p*** **<.001**, η² = .002	*F(1*,* 9234) = 42.727*, ***p*** **<.001**, η² = .004
Pattern	94.16 ± 21.90	89.81 ± 22.04	*F(1*,* 8321)* = 22.29, ***p*** **<.001**, η² = .002	*F(1*,* 9220) = 38.049*, ***p*** **<.001**, η² = .005
**White Matter Microstructure**				
**Fractional Anisotropy (FA)**				
FA Mean	0.475 ± 0.017	0.472 ± 0.019	*F(1*,* 8438)* = 18.92, ***p*** **<.001**, η² = .002	*F(1*,* 9340)* = 33.14, ***p*** **<.001**, η² = .004
AF	0.573 ± 0.028	0.569 ± 0.031	*F(1*,* 8438)* = 17.59, ***p*** **<.001**, η² = .002	*F(1*,* 9340)* = 26.26, ***p*** **<.001**, η² = .002
CB	0.383 ± 0.026	0.380 ± 0.027	*F(1*,* 8438)* = 17.38, ***p*** **<.001**, η² = .002	*F(1*,* 9340)* = 18.79, ***p*** **<.001**, η² = .002
CC 1	0.491 ± 0.038	0.484 ± 0.042	*F(1*,* 8436)* = 27.62, ***p*** **<.001**, η² = .003	*F(1*,* 9338)* = 34.04, ***p*** **<.001**, η² = .004
CC 2	0.563 ± 0.028	0.558 ± 0.031	*F(1*,* 8438)* = 20.12, ***p*** **<.001**, η² = .001	*F(1*,* 9340)* = 24.48, ***p*** **<.001**, η² = .003
CC 3	0.597 ± 0.025	0.593 ± 0.028	*F(1*,* 8437)* = 17.22, ***p*** **<.001**, η² = .001	*F(1*,* 9339)* = 23.86, ***p*** **<.001**, η² = .003
CC 4	0.612 ± 0.025	0.609 ± 0.028	*F(1*,* 8438)* = 13.37, ***p*** **=.010**, η² < .001	*F(1*,* 9340)* = 21.96, ***p*** **<.001**, η² = .003
CPC	0.577 ± 0.026	0.573 ± 0.027	*F(1*,* 8434)* = 14.40, ***p*** **=.006**, η² < .001	*F(1*,* 9335)* = 17.15, ***p*** **<.001**, η² = .002
CRF	0.551 ± 0.026	0.547 ± 0.027	*F(1*,* 8438)* = 12.46, ***p*** **=.017**, η² < .001	*F(1*,* 9340)* = 19.21, ***p*** **<.001**, η² = .002
EC	0.550 ± 0.030	0.543 ± 0.056	*F(1*,* 8437)* = 18.27, ***p*** **<.001**, η² = .001	*F(1*,* 9339)* = 30.73, ***p*** **<.001**, η² = .003
EmC	0.558 ± 0.031	0.552 ± 0.034	*F(1*,* 8437)* = 18.88, ***p*** **<.001**, η² = .002	*F(1*,* 9338)* = 30.93, ***p*** **<.001**, η² = .003
IOFF	0.613 ± 0.030	0.607 ± 0.033	*F(1*,* 8437)* = 18.65, ***p*** **<.001**, η² = .001	*F(1*,* 9338)* = 35.15, ***p*** **<.001**, η² = .004
PLIC	0.479 ± 0.030	0.473 ± 0.031	*F(1*,* 8438)* = 18.26, ***p*** **<.001**, η² = .002	*F(1*,* 9340)* = 29.10, ***p*** **<.001**, η² = .003
SF	0.362 ± 0.020	0.359 ± 0.021	*F(1*,* 8438)* = 20.66, ***p*** **<.001**, η² = .002	*F(1*,* 9340)* = 25.58, ***p*** **<.001**, η² = .003
SLF III.	0.476 ± 0.028	0.473 ± 0.031	*F(1*,* 8438)* = 13.83, ***p*** **=.008**, η² = .001	*F(1*,* 9339)* = 18.69 ***p*** **=.012**, η² = .002
Sup-F	0.404 ± 0.019	0.400 ± 0.021	*F(1*,* 8438)* = 25.17, *p* <.001, η² = .002	*F(1*,* 9340)* = 36.92, ***p*** **<.001**, η² = .004
TF	0.420 ± 0.019	0.416 ± 0.021	*F(1*,* 8438)* = 24.94, ***p*** **<.001**, η² = .002	*F(1*,* 9340)* = 35.56, ***p*** **<.001**, η² = .004
TP	0.424 ± 0.021	0.421 ± 0.022	*F(1*,* 8438)* = 11.42, ***p*** **=.029**, η² = .001	*F(1*,* 9340)* = 17.49, ***p*** **<.001**, η² = .002
UF	0.388 ± 0.031	0.383 ± 0.032	*F(1*,* 8438)* = 20.26, ***p*** **<.001**, η² = .002	*F(1*,* 9340)* = 22.25, ***p*** **<.001**, η² < .001
**Axial Diffusivity (AD)**				
AD Mean	0.997 ± 0.024	0.995 ± 0.024	*F(1*,* 8438)* = 4.34, ***p*** **=.037**, η² < .001	*F(1*,* 9340)* = 79.16, ***p*** **=.005**, η² < .001
CB	0.959 ± 0.032	0.955 ± 0.033	*F(1*,* 8438)* = 15.57, ***p*** **<.001**, η² < .001	*F(1*,* 9340)* = 15.13, ***p*** **=.004**, η² = .002
CC 1	1.048 ± 0.046	1.040 ± 0.048	*F(1*,* 8436)* = 26.83, ***p*** **<.001**, η² = .002	*F(1*,* 9338)* = 29.53, ***p*** **<.001**, η² = .003
UF	0.927 ± 0.028	0.923 ± 0.029	*F(1*,* 8438)* = 13.28, ***p*** **=.011**, η² < .001	*F(1*,* 9340)* = 14.93, ***p*** **=.005**, η² = .002
**Radial Diffusivity (RD)**				
RD Mean	0.416 ± 0.014	0.419 ± 0.015	*F(1*,* 8438)* = 18.77, ***p*** **<.001**, η² = .002	*F(1*,* 9340)* = 29.93, ***p*** **<.001**, η² = .003
AF	0.367 ± 0.020	0.370 ± 0.023	*F(1*,* 8438)* = 16.33, ***p*** **<.001**, η² = .002	*F(1*,* 9340)* = 24.94, ***p*** **<.001**, η² = .003
CC 1	0.434 ± 0.029	0.438 ± 0.031	*F(1*,* 8436)* = 11.69, ***p*** **=.025**, η² = .001	*F(1*,* 9338)* = 15.53, ***p*** **<.001**, η² = .002
CC 2	0.393 ± 0.020	0.396 ± 0.022	*F(1*,* 8438)* = 14.41, ***p*** **=.006**, η² = .002	*F(1*,* 9340)* = 16.67, ***p*** **<.001**, η² = .002
CC 3	0.370 ± 0.019	0.373 ± 0.021	*F(1*,* 8437)* = 16.92, ***p*** **<.001**, η² = .002	*F(1*,* 9339)* = 22.84, ***p*** **<.001**, η² = .002
CC 4	0.357 ± 0.020	0.361 ± 0.023	*F(1*,* 8438)* = 21.55, ***p*** **<.001**, η² = .002	*F(1*,* 9340)* = 33.77, ***p*** **<.001**, η² = .004
CPC	0.355 ± 0.015	0.356 ± 0.016	*F(1*,* 8429)* = 11.15, ***p*** **=.034**, η² = .001	*F(1*,* 9335)* = 10.75, ***p*** **=.042**, η² = .001
EC	0.386 ± 0.020	0.389 ± 0.021	*F(1*,* 8437)* = 11.57, ***p*** **=.027**, η² = .002	*F(1*,* 9339)* = 21.94, ***p*** **<.001**, η² = .002
EmC	0.383 ± 0.023	0.386 ± 0.025	*F(1*,* 8437)* = 13.64, ***p*** **=.009**, η² = .002	*F(1*,* 9338)* = 23.83, ***p*** **<.001**, η² = .003
IOFF	0.354 ± 0.022	0.358 ± 0.024	*F(1*,* 8437)* = 12.74, ***p*** **=.014**, η² = .002	*F(1*,* 9338)* = 27.22, ***p*** **<.001**, η² = .003
MdLF	0.426 ± 0.019	0.429 ± 0.020	*F(1*,* 8438)* = 12.52, ***p*** **=.016**, η² = .001	*F(1*,* 9340)* = 28.84, ***p*** **<.001**, η² = .003
PLIC	0.426 ± 0.021	0.429 ± 0.022	*F(1*,* 8438)* = 14.01, ***p*** **=.007**, η² = .002	*F(1*,* 9340)* = 23.40, ***p*** **<.001**, η² = .002
SF	0.461 ± 0.016	0.463 ± 0.016	*F(1*,* 8438)* = 13.34, ***p*** **=.010**, η² = .001	*F(1*,* 9340)* = 16.81, ***p*** **<.001**, η² = .002
SLF III.	0.424 ± 0.019	0.427 ± 0.022	*F(1*,* 8438)* = 13.87, ***p*** **=.008**, η² = .001	*F(1*,* 9339)* = 17.80, ***p*** **<.001**, η² = .002
SLF II.	0.406 ± 0.021	0.409 ± 0.023	*F(1*,* 8437)* = 11.27, ***p*** **=.033**, η² = .001	*F(1*,* 9337)* = 17.00, ***p*** **=.002**, η² = .002
Sup- F	0.450 ± 0.015	0.452 ± 0.016	*F(1*,* 8438)* = 18.11, ***p*** **<.001**, η² = .002	*F(1*,* 9340)* = 25.56, ***p*** **<.001**, η² = .003
Sup-OT	0.436 ± 0.017	0.439 ± 0.018	*F(1*,* 8438)* = 11.50, ***p*** **=.028**, η² = .001	*F(1*,* 9340)* = 21.97, ***p*** **<.001**, η² = .002
Sup-O	0.473 ± 0.022	0.476 ± 0.024	*F(1*,* 8436)* = 14.05, ***p*** **=.007**, η² = .002	*F(1*,* 9338)* = 20.41, ***p*** **<.001**, η² = .002
Sup-PO	0.472 ± 0.021	0.475 ± 0.021	*F(1*,* 8438)* = 14.73, ***p*** **=.005**, η² = .002	*F(1*,* 9340)* = 21.37, ***p*** **<.001**, η² = .002
Sup-PT	0.432 ± 0.018	0.435 ± 0.020	*F(1*,* 8438)* = 11.10, ***p*** **=.035**, η² = .001	*F(1*,* 9340)* = 21.79, ***p*** **<.001**, η² = .002
Sup-P	0.442 ± 0.018	0.445 ± 0.019	*F(1*,* 8438)* = 12.47, ***p*** **=.017**, η² = .001	*F(1*,* 9340)* = 21.00, ***p*** **<.001**, η² = .002
TF	0.438 ± 0.013	0.440 ± 0.015	*F(1*,* 8438)* = 15.16, ***p*** **<.001**, η² = .002	*F(1*,* 9340)* = 21.25, ***p*** **<.001**, η² = .002
TO	0.411 ± 0.023	0.414 ± 0.025	*F(1*,* 8438)* = 10.97, ***p*** **=.037**, η² < .001	*F(1*,* 9340)* = 17.23, ***p*** **=.001**, η² = .002
TP	0.442 ± 0.017	0.444 ± 0.018	*F(1*,* 8438)* = 10.55, ***p*** **=.047**, η² = .001	*F(1*,* 9340)* = 13.78, ***p*** **=.008**, η² = .001
UF	0.478 ± 0.021	0.480 ± 0.022	*F(1*,* 8438)* = 13.05, ***p*** **=.012**, η² = .002	*F(1*,* 9340)* = 14.30, ***p*** **=.006**, η² = .002

All p-values were multiplied by the number of investigated variables (by 10 for behavior, by 9 for cognition, and by 40 for whitematter microstructure). Here, only white matter tracts that were statistically significant between groups for calculations both with andwithout covarying for age, sex, study site, handedness, race, and total family income, are listed.

Abbreviations. AF = arcuate fasciculus, CB = cingulum bundle, CC = corpus callosum, CPC = cortico-ponto cerebellar, CRF = corona-radiata-frontal, EC = external capsule, EmC = extreme capsule, IOFF = inferior occipito-frontal fasciculus, MdLF = middle longitudinal fasciculus, PLIC = posterior limb of internal capsule, SF = striato-frontal, SLF = superior longitudinal fasciculus, Sup-F = superficial frontal, Sup-OT = superior occipito-temporal, Sup-P = superior parietal, Sup-PO = superficial parieto-occpital, Sup-P = superficial occipital, Sup-OT = superficial occipital-temporal, TF = thalamo-frontal, TO = thalamo-occipital, TP = thalamo-parietal, UF = uncinate fasciculus

### Odds ratios

**Fig. 2:**
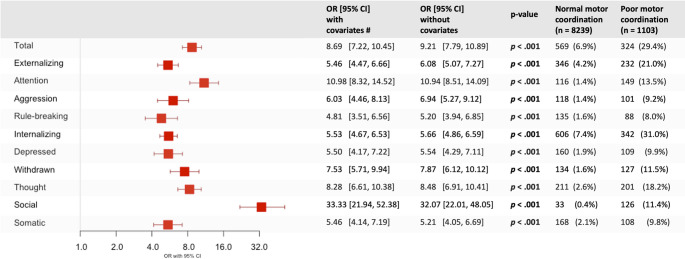
Increased risk for behavioral difficulties in the group with poor motor coordination compared to the group with normal motor coordination illustrated as Odds Ratios and 95% confidence intervals Note. # Includes the adjustment for the following covariates: age, sex, study site, race, handedness, and total family income. Abbreviations. CI = confidence interval, OR = odds ratio

In this sample, 29.4% of children with poor parent-reported motor coordination, and 6.9% of children with normal motor coordination were reported to demonstrate behavioral difficulties (Total Summary Score) (Figure 2). Calculations with and without covariate corrections resulted in similar results. In detail, children with poor motor coordination had 8.69 higher odds of exhibiting behavioral difficulties (Total Summary Score) compared to the group with normal motor coordination. The odds were 5.46 times higher for the Externalizing Summary Score, 5.53 times higher for the Internalizing Summary Score, 10.98 times higher for Attention Problems, 4.81 times higher for Rule-breaking Behavior, 6.03 times higher for Aggressive Behavior, 5.50 times higher for Anxious/Depressed Behavior, 7.53 times higher for Withdrawal Behavior, 8.28 times higher for Thought Problems, 33.33 times higher for Social Problems, and 5.46 times higher for Somatic Problems (all three summary scores and eight sub-scores* p* <.001).

In summary, this study revealed a significantly higher odds of behavioral difficulties in children with poor compared to those with normal motor coordination.

### Group differences in cognitive functioning

Between-group assessments (normal/poor motor coordination) covarying for sex, study site, handedness, race, and total family income revealed statistically significant lower performance in the total (*p* <.001), fluid composite (*p* <.001), but not the crystal composite score (*p* >.999) in the group with poor parent-reported motor coordination compared to those with normal motor coordination. Moreover, children with poor parent-reported coordination performed significantly worse in all individual tests (*p* <.001; Table 2; Fig. 1) except for the Picture Vocabulary Test (*p* >.999), compared to children with normal coordination. Between-group differences in the crystal composite score with worse performance in the group with poor parent-reported motor coordination were only significant without covarying for sex, study site, handedness, race, and total family income.

### Group differences in white matter microstructure

Between-group assessments (normal/poor coordination) covarying for sex, age, handedness, race, and total family income revealed statistically significant lower whole-brain average FA and AD, and higher whole-brain average RD in the group with poor coordination (Figure 1). Significant differences were found in large parts of the white matter as listed in Table 2. Calculations with and without covariates yielded similar results.

### Mediation analysis

Average FA mediated 0.3% (95%-CI [.0003;.01]) of the relationship between poor parent-reported motor coordination and behavioral difficulties as assessed by the Total Summary Score as part of the CBCL (*p*_ACME_ =.028). Both the total effect (direct effect of motor coordination plus indirect effect over FA) and the direct effect (without the indirect effect) were significant (both *p* <.001), indicating a partial mediation effect. Average FA mediated 8.12% (95%-CI [.0456;.12]) of the relationship between poor parent-reported motor coordination and cognitive functioning as assessed by the Total Composite Score assessed by the NIH toolbox (*p*_ACME_ <.001). Again, both total and direct effect were significant (both *p* <.001), indicating a partial mediation effect.

To provide a more detailed overview, Supplementary Tables S1 and S2 list the mediation results for fractional anisotropy across all significant white-matter tracts identified in the group-difference analyses, presented separately for the CBCL Total Summary Score and the NIH Toolbox Total Composite Score models.

## Discussion

Results from this large population-based study with more than 9000 children, shows that children with poor parent-reported motor coordination are more likely to experience behavioral and cognitive difficulties. Moreover, these children demonstrate alterations in white matter microstructure, particularly lower FA and higher RD which are indicative of potential differences in myelination. Rather than reflecting a motor-specific pathway, these white matter alterations may represent an *epiphenomenon* of a broader neurodevelopmental profile in which motor, behavioral, and cognitive difficulties commonly co-occur. These findings align with previous research and suggest that motor coordination can serve as a useful early marker for broader neurodevelopmental risk.

In this sample, 11.8% of children displayed poor parent-reported motor coordination which is in line with previous epidemiological studies estimating that 5 to 15% of school-aged children demonstrate poor motor coordination [31]. Moreover, we found children with poor parent-reported motor coordination more likely to be younger, male, from families with low socioeconomic status, to have higher BMI, ambidextrous handedness, and identify as Black. The latter finding should be treated with caution as race was significantly correlated with parental income reflecting socioeconomic status, thus potentially attributable to the same underlying factor. This is in line with preliminary evidence stemming from smaller samples reporting that younger children are more likely affected than older children [32], children with higher BMI are more likely affected than children with lower body weight [33], boys are more likely affected than girls [34], and children from families with lower socioeconomic status are more likely affected than children from high socioeconomic status [35]. We now confirm these associations in a large and representative sample of almost 10,000 children.

### Behavior

Across the entire sample, the prevalence of behavioral difficulties is 9.56%. This is very close to the 9.6% prevalence of behavioral difficulties among 8-9-year-old children reported by a previous study [36]. However, when we separated the cohort based on motor function, we found children with poor parent-reported motor coordination to have higher odds of exhibiting behavioral difficulties compared to children with normal motor coordination. Only 6.9% of children with normal coordination, but 29.4% of children with poor parent-reported motor coordination demonstrated behavioral difficulties based on the CBCL total score.

This is in line with previous studies reporting that motor coordination may be associated and even predictive of behavior [37, 38]. Of note, while the previous study used neurological examination to assess motor coordination, here we used parent-reported motor coordination and found similar results. 

Children with poor parent-reported motor coordination showed increased odds of all dimensions of behavioral difficulties. The elaborated environmental stress hypothesis suggests that poor motor coordination is indirectly associated with internalizing behavior [9]. This theoretical framework proposes that poor motor coordination leads to psychosocial changes in the individual’s environment such as lower social support or lower self-competence which subsequently leads to increased internalizing behavior. Also externalizing behavior has shown to play a major role in this context and be linked to motor coordination. Our findings of social problems also align with a study investigating 234 children (mean age 9 years 7 months) that found children with poor motor skills as assessed using the McCarron Assessment of Neuromuscular Development, to have limited empathic ability and affected social behavior [39].

### Cognitive functioning

Children with poor parent-reported motor coordination performed worse in tests assessing cognitive function compared to children with normal motor coordination. In 1953, Piaget already suggested that the development of motor skills in children is associated with the development of cognitive functioning [40]. This theory has been confirmed by later studies investigating large cohorts of children showing that investigated motor coordination in children is associated with cognitive functioning [41]. Interestingly, evidence shows similar brain structures such as the prefrontal cortex and the cerebellum to be involved in the development of motor coordination and cognitive functioning [42]. Our finding thus further supports the hypothesis that motor and cognitive development are highly inter-related and mature along similar trajectories. After covariate correction, we detected lower performance in children with poor motor skills in tests measuring fluid cognition, but not in tests measuring crystallized cognition. It is assumed that fluid cognition can be altered in response to alterations in biological processes, while crystallized cognition is rather stable over time [43].

### White matter microstructure

Using dMRI, we found lower FA and AD, as well as higher RD in several white matter tracts of the brain in children with poor parent-reported motor coordination. These findings align with recent work from our group where we investigated typically developing adolescents aged 13 to 16 years [44]. In this study we assessed neurological soft signs, subtle alterations in motor functioning as detected during a pediatric neurological examination. Here, we found lower FA and higher RD in clusters spanning similar white matter tracts as reported in the previous study based on a different sample [44].

FA is considered a summary measure of white matter microstructure that has been shown to be lower in several psychiatric disorders [45]. It is, however, considered rather unspecific regarding alterations on a cellular level. As suggested by Jones et al. (2013), FA reflects a voxel-averaged mixture of influences—including crossing fibers, orientation dispersion, axonal density, myelination, and partial-volume effects—and therefore should not be overinterpreted [46]. For a more specific interpretation, we have retrieved additional diffusion parameters such as AD and RD. Higher radial diffusivity is commonly interpreted as alteration in myelination. Lower AD is purported to reflect axonal alteration [47]. In most tracts, we found lower FA and higher RD in the group of children with poor motor coordination. In some posterior and superficial tracts, we only found higher RD, but not lower FA. The combination of increased FA and decreased RD is considered to reflect myelination, a hallmark process of brain development [48]. It is assumed that myelination ensures high speed and efficiency of information flow between brain regions and thus optimizes brain function. Thus, our finding of widespread lower FA and higher RD in several white matter tracts of the brain suggests alterations or delays in myelination in the group of children with poor motor coordination. In a few white matter tracts, we also found lower AD. This finding is in alignment with a previous study that found lower AD in a group of children with DCD compared to a typically developing group [49]. Based on this finding, it can be hypothesized that in some tracts, alterations of the axon in addition to the myelin sheath are present.

 Alterations in white matter microstructure have previously been related to alterations in behavioral and cognitive functioning in children and adolescents (for review see [50]). One study, for example, found a statistically significant correlation between lower FA in the CC and CB and higher anxiety and depression scores in the CBCL which is similar to our findings [51]. Moreover, studies found children with lower cognitive functioning to have lower FA and higher RD in the SLF [52], the CC [53], lower FA in the IFOF and higher MD in the ILF and UF [54] which is further supported by our findings. 

Taken together, children with poor parent-reported motor coordination show signs of altered white matter microstructure in tracts that play a major role in motor, behavioral, and cognitive functioning. These alterations may primarily reflect alterations in myelination and to a smaller extent also alterations in axonal development.

### White matter microstructure as mediator 

We found that FA mediates approximately 8% of the relationship between motor coordination and a total composite score of cognition. The mediation effect of FA on the relationship between motor coordination and behavior, albeit being statistically significant, was small. A previous study on young adults showed that FA mediates the association between motor and cognitive performance [55]. However, there is limited research on this mediation in children. Our findings suggest that white matter development is crucial for motor coordination, problem behavior, and cognitive functioning. Nonetheless, it only partly explains their association. Instead, this relationship is likely influenced by a complex combination of biological and environmental factors.

## Limitations

There are limitations of this study that need to be taken into consideration when interpreting the results. First, the ABCD study does not include a pediatric neurological examination which is considered the gold-standard for assessing motor coordination in children. The classification of poor versus normal motor coordination in this study is based on item 62 of the CBCL asking parents for a categorical report of their child’s motor coordination. Evidence from a previous study comparing the use of CBCL item 62 with the outcome derived from a neurological examination called *McCarron Assessment of Neuromuscular Development* showed a high sensitivity but low specificity between the two assessments [56]. Based on this finding, the identification of poor motor coordination through accessible parent-report tools should not replace gold-standard measurements but potentially can facilitate early screening for broader developmental challenges. In case of concerning signs in the screening assessment, standardized tools such as the Movement Assessment Battery for Children (MABC-3) should follow. Of note, the findings presented in this study align closely with prior work reporting differences in overlapping white-matter pathways between children with and without motor coordination problems based on a standardized neurological examination [44]. This convergence across methods (parent-reported screening vs. gold-standard neurological exam) with similar findings mitigates the limitation inherent in relying on a single CBCL item.

Second, groups with normal coordination and poor coordination were significantly different regarding age, BMI, sex, handedness, total family income, and race. Even though, for transparency, we performed two different analyses for all findings by controlling for all significant confounders, and reporting uncontrolled findings, we do not know with certainty to what extent these confounders may have influenced the results. However, using a population-based approach allowed us to identify demographical characteristics of children that may be at an increased risk for motor coordination, behavioral and cognitive difficulties.

Third, the direction of causality between motor coordination, problem behavior, cognitive functioning and underlying white matter microstructure is not yet fully understood. Our mediation analysis revealed that white matter microstructure partially explains the link between motor coordination and behavior/cognition. This provides a starting point to explore causality.

Finally, while effect sizes ranged from very small to large—limiting the magnitude of some associations—their consistent significance in this well-powered sample provides precise and replicable evidence for relationships among motor coordination, behavioral and cognitive outcomes, and white matter microstructure.

## Conclusion

Based on a large sample size, this study provides evidence that children with male sex, higher BMI, ambidextrous handedness, from a low socio-economic status and identified as Black are at a higher risk for poor motor coordination. Compared to children with normal motor coordination, children with poor motor coordination are more likely to display difficulties in behavior and cognition. Further, children with poor motor coordination showed alterations in white matter microstructure suggestive of alterations in myelination. Taken together, our results emphasize the importance of early identification of motor coordination difficulties, not only as a standalone concern but as a potential window into broader neurodevelopmental challenges, including behavior and cognition. This highlights the need for multidisciplinary approaches in pediatric assessment and care.

## Supplementary Information

Below is the link to the electronic supplementary material.

Supplementary Material 1 (DOCX. 25.9 KB)

## Data Availability

A data use certificate has been signed between the authors and NIHM Data Archive. Data used in the preparation of this article were obtained from the Adolescent Brain Cognitive Development (ABCD) Study (https://abcdstudy.org), held in the NIMH Data Archive (NDA). This is a multisite, longitudinal study designed to recruit more than 10,000 children aged 9-10 and follow them over 10 years into early adulthood. The ABCD Study^®^ is supported by the National Institutes of Health and additional federal partners under award numbers U01DA041048, U01DA050989, U01DA051016, U01DA041022, U01DA051018, U01DA051037, U01DA050987, U01DA041174, U01DA041106, U01DA041117, U01DA041028, U01DA041134, U01DA050988, U01DA051039, U01DA041156, U01DA041025, U01DA041120, U01DA051038, U01DA041148, U01DA041093, U01DA041089, U24DA041123, U24DA041147. A full list of supporters is available at [https://abcdstudy.org/federal-partners.html](https:/abcdstudy.org/federal-partners.html). A listing of participating sites and a complete listing of the study investigators can be found at [https://abcdstudy.org/consortium\_members/](https:/abcdstudy.org/consortium_members). ABCD consortium investigators designed and implemented the study and/or provided data but did not necessarily participate in the analysis or writing of this report. This manuscript reflects the views of the authors and may not reflect the opinions or views of the NIH or ABCD consortium investigators.
