# Supplementary material for: The association between motor coordination, behavior, cognition and brain structure in children: an ABCD study
Source: Eur Child Adolesc Psychiatry. 2026 Apr 6;35(6):1983–95. doi: 10.1007/s00787-025-02931-2 (PMC13337589; doi:10.1007/s00787-025-02931-2)
Supplement: Supplementary file 1 — Supplementary Material 1 (DOCX. 25.9 KB) [file 787_2025_2931_MOESM1_ESM.docx]

S1: Mediation results for fractional anisotropy across significant white matter tracts for the NIH Toolbox Total Composite Score

| Mediator | Indirect (ACME) | p(ACME) | Direct (ADE) | p(ADE) | Total effect (τ) | p(Total) | Proportion mediated | p(Prop) |
| --- | --- | --- | --- | --- | --- | --- | --- | --- |
| FA Mean | -0.37 [-0.53, -0.21] | < .001 | -4.18 [-5.35, -3.08] | < .001 | -4.55 [-5.68, -3.45] | < .001 | 0.08 [0.05, 0.12] | < .001 |
| AF | -0.36 [-0.54, -0.20] | < .001 | -4.19 [-5.25, -3.07] | < .001 | -4.55 [-5.59, -3.47] | < .001 | 0.08 [0.04, 0.12] | < .001 |
| CB | -0.18 [-0.30, -0.08] | < .001 | -4.37 [-5.50, -3.23] | < .001 | -4.55 [-5.71, -3.42] | < .001 | 0.04 [0.02, 0.07] | < .001 |
| CC 1 | -0.26 [-0.37, -0.15] | < .001 | -4.30 [-5.47, -3.23] | < .001 | -4.56 [-5.74, -3.49] | < .001 | 0.06 [0.03, 0.09] | < .001 |
| CC 2 | -0.31 [-0.48, -0.16] | < .001 | -4.24 [-5.43, -3.12] | < .001 | -4.55 [-5.71, -3.42] | < .001 | 0.07 [0.04, 0.11] | < .001 |
| CC 3 | -0.35 [-0.52, -0.19] | < .001 | -4.20 [-5.28, -3.03] | < .001 | -4.55 [-5.62, -3.36] | < .001 | 0.08 [0.04, 0.12] | < .001 |
| CC 4 | -0.26 [-0.40, -0.13] | < .001 | -4.29 [-5.40, -3.19] | < .001 | -4.55 [-5.68, -3.45] | < .001 | 0.06 [0.03, 0.10] | < .001 |
| CPC | -0.13 [-0.23, -0.06] | < .001 | -4.38 [-5.42, -3.22] | < .001 | -4.52 [-5.54, -3.37] | < .001 | 0.03 [0.01, 0.05] | < .001 |
| CRF | -0.22 [-0.32, -0.11] | < .001 | -4.33 [-5.49, -3.20] | < .001 | -4.55 [-5.70, -3.40] | < .001 | 0.05 [0.02, 0.08] | < .001 |
| EC | -0.39 [-0.56, -0.23] | < .001 | -4.16 [-5.27, -2.99] | < .001 | -4.55 [-5.71, -3.36] | < .001 | 0.09 [0.05, 0.13] | < .001 |
| EmC | -0.30 [-0.44, -0.18] | < .001 | -4.25 [-5.37, -3.12] | < .001 | -4.55 [-5.69, -3.43] | < .001 | 0.07 [0.04, 0.11] | < .001 |
| IOFF | -0.37 [-0.53, -0.23] | < .001 | -4.18 [-5.38, -3.05] | < .001 | -4.55 [-5.73, -3.42] | < .001 | 0.08 [0.05, 0.13] | < .001 |
| PLIC | -0.36 [-0.52, -0.21] | < .001 | -4.19 [-5.24, -2.99] | < .001 | -4.55 [-5.61, -3.34] | < .001 | 0.08 [0.05, 0.13] | < .001 |
| SF | -0.30 [-0.43, -0.16] | < .001 | -4.25 [-5.40, -3.20] | < .001 | -4.55 [-5.71, -3.50] | < .001 | 0.07 [0.04, 0.10] | < .001 |
| SLF III. | -0.19 [-0.31, -0.09] | .002 | -4.36 [-5.45, -3.21] | < .001 | -4.55 [-5.65, -3.42] | < .001 | 0.04 [0.02, 0.07] | .002 |
| Sup-F | -0.37 [-0.53, -0.23] | < .001 | -4.18 [-5.32, -3.03] | < .001 | -4.55 [-5.70, -3.37] | < .001 | 0.08 [0.05, 0.13] | < .001 |
| TF | -0.43 [-0.60, -0.27] | < .001 | -4.12 [-5.28, -3.03] | < .001 | -4.55 [-5.71, -3.47] | < .001 | 0.09 [0.06, 0.14] | < .001 |
| TP | -0.32 [-0.52, -0.16] | < .001 | -4.23 [-5.40, -3.14] | < .001 | -4.55 [-5.73, -3.47] | < .001 | 0.07 [0.03, 0.12] | < .001 |
| UF | -0.22 [-0.33, -0.12] | < .001 | -4.33 [-5.50, -3.16] | < .001 | -4.55 [-5.72, -3.39] | < .001 | 0.05 [0.03, 0.08] | < .001 |

Abbreviations. AF = arcuate fasciculus, CB = cingulum bundle, CC = corpus callosum, CPC = cortico-ponto-cerebellar, CRF = corona-radiata-frontal, EC = external capsule, EmC = extreme capsule, IOFF = inferior occipito-frontal fasciculus, MdLF = middle longitudinal fasciculus, PLIC = posterior limb of internal capsule, SF = striato-frontal, SLF = superior longitudinal fasciculus, Sup-F = superficial frontal, Sup-OT = superior occipito-temporal, Sup-P = superior parietal, Sup-PO = superficial parieto-occpital, Sup-P = superficial occipital, Sup-OT = superficial occipital-temporal, TF = thalamo-frontal, TO = thalamo-occipital, TP = thalamo-parietal, UF = uncinate fasciculus

S2: Mediation results for fractional anisotropy across significant white matter tracts for the CBCL Total Summary Score

| Mediator | Indirect (ACME) | p(ACME) | Direct (ADE) | p(ADE) | Total effect (τ) | p(Total) | Proportion mediated | p(Prop) |
| --- | --- | --- | --- | --- | --- | --- | --- | --- |
| FA Mean | 0.04 [0.00, 0.09] | .028 | 13.67 [13.02, 14.29] | < .001 | 13.71 [13.06, 14.34] | < .001 | 0.00 [0.00, 0.01] | .028 |
| AF | 0.03 [-0.00, 0.08] | .052 | 13.67 [13.04, 14.32] | < .001 | 13.71 [13.08, 14.34] | < .001 | 0.00 [-0.00, 0.01] | .052 |
| CB | 0.02 [-0.01, 0.06] | .170 | 13.69 [13.03, 14.25] | < .001 | 13.71 [13.05, 14.29] | < .001 | 0.00 [-0.00, 0.00] | .170 |
| CC 1 | 0.01 [-0.03, 0.05] | .658 | 13.69 [13.06, 14.32] | < .001 | 13.70 [13.07, 14.32] | < .001 | 0.00 [-0.00, 0.00] | .658 |
| CC 2 | 0.02 [-0.01, 0.06] | .210 | 13.68 [13.09, 14.33] | < .001 | 13.71 [13.12, 14.36] | < .001 | 0.00 [-0.00, 0.00] | .210 |
| CC 3 | 0.03 [-0.00, 0.07] | .080 | 13.68 [13.04, 14.29] | < .001 | 13.71 [13.06, 14.33] | < .001 | 0.00 [-0.00, 0.01] | .080 |
| CC 4 | 0.03 [0.00, 0.07] | .048 | 13.67 [13.00, 14.26] | < .001 | 13.71 [13.04, 14.30] | < .001 | 0.00 [0.00, 0.01] | .048 |
| CPC | 0.01 [-0.02, 0.04] | .454 | 13.69 [13.05, 14.36] | < .001 | 13.70 [13.06, 14.37] | < .001 | 0.00 [-0.00, 0.00] | .454 |
| CRF | 0.03 [0.00, 0.06] | .038 | 13.68 [13.07, 14.29] | < .001 | 13.71 [13.10, 14.32] | < .001 | 0.00 [0.00, 0.00] | .038 |
| EC | 0.05 [0.01, 0.10] | .008 | 13.65 [13.01, 14.27] | < .001 | 13.70 [13.07, 14.33] | < .001 | 0.00 [0.00, 0.01] | .008 |
| EmC | 0.05 [0.01, 0.09] | .016 | 13.65 [12.99, 14.26] | < .001 | 13.70 [13.03, 14.30] | < .001 | 0.00 [0.00, 0.01] | .016 |
| IOFF | 0.07 [0.03, 0.12] | < .001 | 13.63 [13.00, 14.25] | < .001 | 13.70 [13.07, 14.33] | < .001 | 0.01 [0.00, 0.01] | < .001 |
| PLIC | 0.06 [0.02, 0.10] | .006 | 13.65 [13.00, 14.27] | < .001 | 13.71 [13.07, 14.33] | < .001 | 0.00 [0.00, 0.01] | .006 |
| SF | 0.05 [0.01, 0.09] | .012 | 13.66 [13.01, 14.29] | < .001 | 13.71 [13.06, 14.33] | < .001 | 0.00 [0.00, 0.01] | .012 |
| SLF III. | 0.03 [-0.00, 0.06] | .096 | 13.68 [13.04, 14.33] | < .001 | 13.71 [13.09, 14.35] | < .001 | 0.00 [-0.00, 0.00] | .096 |
| Sup-F | 0.05 [0.00, 0.09] | .030 | 13.66 [13.02, 14.33] | < .001 | 13.71 [13.08, 14.37] | < .001 | 0.00 [0.00, 0.01] | .030 |
| TF | 0.07 [0.03, 0.13] | < .001 | 13.63 [13.00, 14.21] | < .001 | 13.71 [13.08, 14.30] | < .001 | 0.01 [0.00, 0.01] | < .001 |
| TP | 0.04 [0.01, 0.08] | .012 | 13.67 [13.03, 14.32] | < .001 | 13.71 [13.07, 14.36] | < .001 | 0.00 [0.00, 0.01] | .012 |
| UF | 0.01 [-0.02, 0.04] | .440 | 13.70 [13.06, 14.32] | < .001 | 13.71 [13.08, 14.34] | < .001 | 0.00 [-0.00, 0.00] | .440 |

Abbreviations. AF = arcuate fasciculus, CB = cingulum bundle, CC = corpus callosum, CPC = cortico-ponto-cerebellar, CRF = corona-radiata-frontal, EC = external capsule, EmC = extreme capsule, IOFF = inferior occipito-frontal fasciculus, MdLF = middle longitudinal fasciculus, PLIC = posterior limb of internal capsule, SF = striato-frontal, SLF = superior longitudinal fasciculus, Sup-F = superficial frontal, Sup-OT = superior occipito-temporal, Sup-P = superior parietal, Sup-PO = superficial parieto-occpital, Sup-P = superficial occipital, Sup-OT = superficial occipital-temporal, TF = thalamo-frontal, TO = thalamo-occipital, TP = thalamo-parietal, UF = uncinate fasciculus
